# Simulated Keratometry Versus Total Keratometry: Separate Evaluations in 932 Eyes with Prior Myopic LASIK and 372 Eyes with Prior Radial Keratotomy

**DOI:** 10.3390/jcm15218749

**Published:** 2026-11-09

**Authors:** Qingying Yang, Wenwen He, Yurui Pan, Yi Lu, Xiangjia Zhu

**Affiliations:** Department of Ophthalmology, Shanghai Key Laboratory of Visual Impairment and Restoration, Eye and ENT Hospital, Fudan University, Shanghai 200031, China

**Keywords:** cataract surgery, laser-assisted in situ keratomileusis, radial keratotomy, keratometry, IOLMaster 700

## Abstract

**Objectives:** To evaluate simulated keratometry (SimK) and total keratometry (TK) in eyes with prior myopic LASIK and radial keratotomy (RK), separately. **Methods:** Patients scheduled for cataract surgery with prior myopic LASIK or RK were included for separate analysis. Ocular biometry was performed using IOLMaster 700 (Carl Zeiss Meditec AG, Jena, Germany). Descriptive statistics for SimK and TK, their difference, correlation, and agreement were evaluated. The factors influencing the difference between SimK and TK were investigated. **Results:** In total, 932 eyes with prior myopic LASIK and 372 eyes with prior RK were analyzed. Compared to TK, SimK was significantly higher by +0.61 D (95% CI, +0.60 to +0.63 D) in eyes with prior myopic LASIK but significantly lower by −0.13 D (95% CI, −0.15 to −0.11 D) in those with prior RK (both *p* < 0.001). SimK and TK were strongly correlated in both groups (r = 0.996 and 0.997, respectively; both *p* < 0.001). The 95% limits of agreement (LoA) were +0.10 to +1.12 D in eyes with prior myopic LASIK and −0.58 to +0.32 D in those with prior RK. The difference between SimK and TK was significantly associated with axial length (AL) and central corneal thickness (CCT) in both groups. **Conclusions:** In eyes with prior myopic LASIK, SimK was higher than TK, while the opposite was observed with prior RK. SimK and TK showed limited agreement in both groups and should not be assumed interchangeable, particularly in eyes after LASIK with longer AL or thinner CCT and eyes after RK with shorter AL or thinner CCT.

## 1. Introduction

With the advancement and adoption of corneal refractive surgery over the past decades, millions of myopic patients have achieved functional independence from corrective lenses [1]. Laser in situ keratomileusis (LASIK) and radial keratotomy (RK) are two representative techniques widely used in their respective eras. However, as this large population of patients with prior myopic LASIK or RK gradually ages and develops cataracts, ensuring optimal visual outcomes after cataract surgery for them has become a considerable challenge [2,3].

A primary source of this challenge is that intraocular lens (IOL) power calculation remains insufficiently reliable in eyes with prior corneal refractive surgery, with formula-dependent prediction errors reported after myopic LASIK [4] and RK [5,6], as well as after other forms of laser vision correction [7,8,9]. A major contributor to this inaccuracy is error in keratometry measurements, as myopic LASIK and RK both permanently alter the biological structure and properties of the cornea, rendering conventional keratometry invalid [10,11,12]. Typically, standard keratometry provides simulated keratometry (SimK), a value derived from the anterior corneal curvature using a keratometric index of 1.3375 [13,14]. More recently, advances in technology have allowed the direct measurement of both anterior and posterior corneal curvatures, consequently enabling the calculation of total keratometry (TK) [15].

Both SimK and TK are key parameters used in IOL power calculation. Studies have shown that TK-based formulas improve prediction accuracy after myopic LASIK, whereas this advantage has not been consistently demonstrated after RK [16,17]. This inconsistency may, in part, reflect an incomplete understanding of the relationship between SimK and TK in eyes with different types of prior refractive surgery. In these eyes, previous studies have mainly focused on agreement between different instruments for keratometry measurements [18,19], rather than on the relationship between SimK and TK obtained with the same device. In normal eyes, in contrast, SimK and TK are strongly associated, although SimK is significantly higher than TK [20]. Whether this relationship is preserved in eyes with prior corneal refractive surgery remains unclear. To the best of our knowledge, the systematic evaluation of SimK and TK in these eyes is lacking, and whether LASIK and RK affect keratometry measurements differently remains insufficiently understood.

Therefore, the purpose of this study was to evaluate SimK and TK measurements by IOLMaster 700 in eyes with prior myopic LASIK and in those with prior RK, separately. This study aimed to characterize the relationship between SimK and TK in each group, and to determine whether this relationship differs between the two procedures, thereby providing a basis for future studies on IOL power calculation for the growing population of aging patients with prior myopic LASIK or RK.

## 2. Methods

### 2.1. Ethical Approval

This cross-sectional study was approved by the Institutional Review Board of Eye and Ear, Nose, and Throat Hospital, Fudan University, and was based on the Shanghai High Myopia Study (ClinicalTrials.gov NCT03062085). All the procedures adhered to the tenets of the Declaration of Helsinki. The requirement for informed consent was waived due to the retrospective and de-identified nature of this study.

### 2.2. Patients

We reviewed the medical records of cataract patients with prior myopic LASIK or RK who were scheduled for phacoemulsification and IOL implantation from January 2021 to June 2025 at Eye and Ear, Nose, and Throat Hospital, Fudan University. The exclusion criteria were as follows: (1) severe ocular diseases such as corneal diseases, glaucoma, strabismus, uveitis, and retinopathies; or (2) a history of ocular trauma or surgery other than LASIK and RK.

### 2.3. Data Collection and Quality Control

For each patient, we collected demographic and clinical information from medical records, including age, gender, eye laterality, and type of prior corneal refractive surgery (myopic LASIK or RK), as well as ocular biometric parameters measured using IOLMaster 700 (Carl Zeiss Meditec AG, Jena, Germany), including axial length (AL), simulated keratometry (SimK), total keratometry (TK), central corneal thickness (CCT), anterior chamber depth (ACD), lens thickness (LT), white-to-white (WTW), angle Alpha, and angle Kappa. All IOLMaster 700 measurements were performed by trained examiners using the same device, according to the manufacturer’s standard non-contact acquisition protocol. Patients were seated, instructed to fixate on the central target, and examined without pupil dilation. The study period spanned from January 2021 to June 2025, during which the device software was updated in routine clinical use; the software versions recorded in the exported data ranged from 1.88.1.64861 to 1.90.38.02. The acquisition protocol remained unchanged throughout the study.

**Figure 1:**
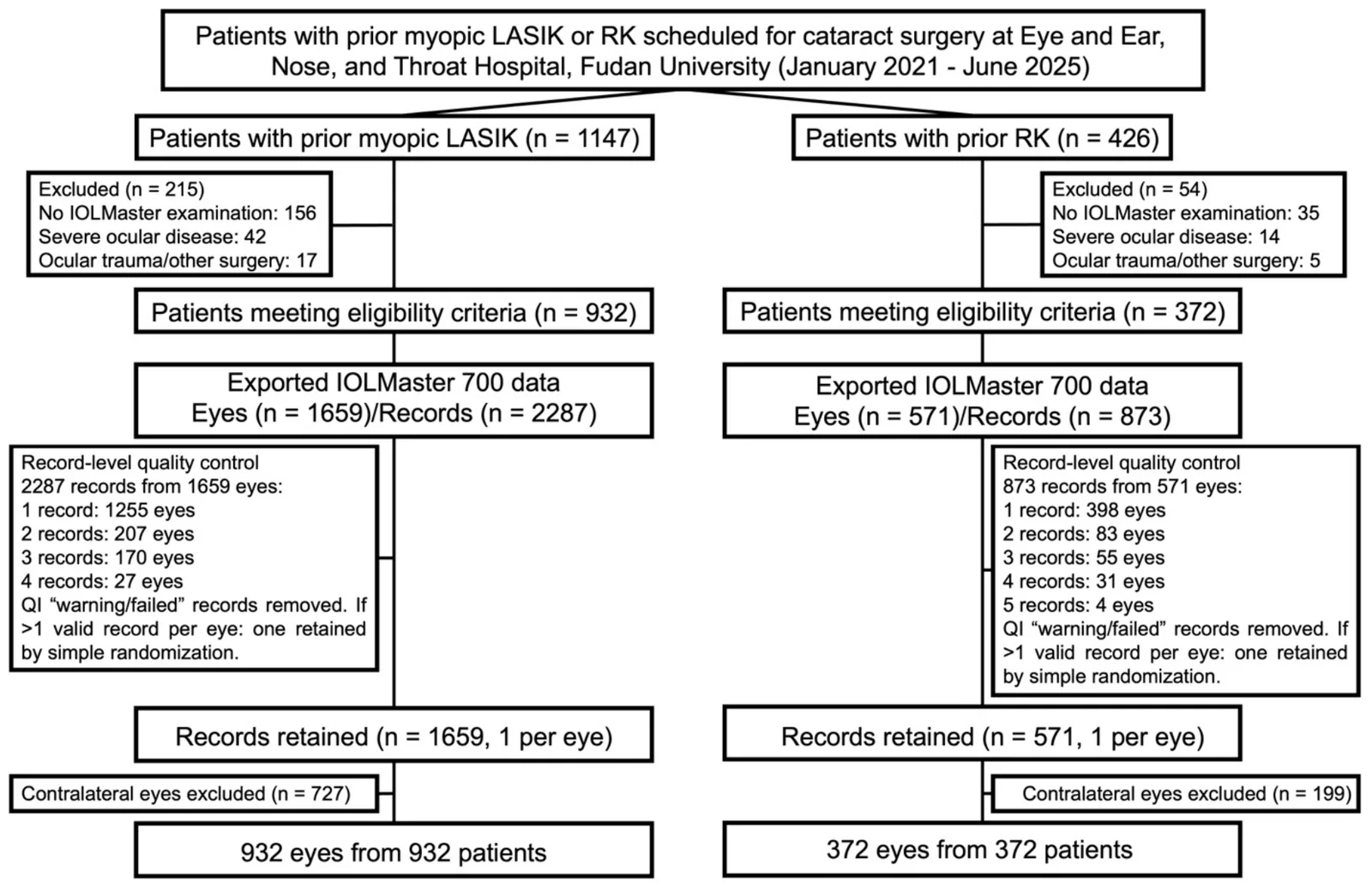
Flow diagram of the screening and exclusion process.

The IOLMaster 700 provides a built-in quality index (QI) for each measurement, categorized as “successful”, “warning”, or “failed”. In routine clinical practice, the number of acquisition attempts per eye was not fixed; examiners generally continued until a valid record with a QI of “successful” for all included biometric parameters was obtained. Consequently, the exported data for each eye contained at least one valid record and could also contain additional records in which one or more parameters had a QI of “warning” or “failed”, as well as, in some cases, more than one valid record. To ensure data quality, records with any included biometric parameter yielding a QI of “warning” or “failed” were excluded. When more than one valid record was available for the same eye, one record was selected by simple randomization: a random number was generated for each candidate record using the RAND() function in Microsoft Excel 2024 (Microsoft Corp., Redmond, WA, USA), and the record with the lowest random value was retained. For patients in whom both eyes were eligible, we selected one eye for inclusion by simple randomization using the same method. No averaging across records was performed. The screening and exclusion process is summarized in Figure 1. The final analysis comprised 932 eyes from 932 patients with prior myopic LASIK and 372 eyes from 372 patients with prior RK.

This retrospective study included patients scheduled for cataract surgery at Eye and Ear, Nose, and Throat Hospital, Fudan University (January 2021–June 2025) with a history of myopic LASIK or radial keratotomy (RK). Patients were divided into the LASIK group (*n* = 1147) and the RK group (*n* = 426). After excluding those without IOLMaster examinations, with severe ocular diseases, or with a history of ocular trauma/other surgeries, IOLMaster 700 biometric data were exported. Record-level quality control was performed by removing “warning/failed” records and randomly retaining one valid record per eye. To ensure statistical independence, contralateral eyes were excluded, resulting in a final inclusion of 932 eyes from 932 patients in the LASIK group and 372 eyes from 372 patients in the RK group.

### 2.4. Statistical Analysis

Statistical analyses were performed using SPSS (version 29.0; IBM Corp, Armonk, NY, USA) and GraphPad Prism (version 9.0; Dotmatics, Boston, MA, USA). The normality of the data was checked using histograms and Q-Q plots, as well as with the Kolmogorov–Smirnov test. Continuous variables (age, AL, SimK, TK, CCT, ACD, LT, WTW, angle Alpha, and angle Kappa) were presented as mean ± standard deviation (SD), and categorical variables (gender and eye laterality) were expressed as numbers and frequencies (%). A two-sided *p*-value < 0.05 was considered statistically significant. Within each group, SimK and TK were compared using a paired *t*-test. The relationship and agreement between SimK and TK were evaluated by Pearson’s correlation analysis and Bland–Altman analysis, respectively. Proportional bias was formally assessed by regressing the difference between SimK and TK (SimK−TK) on their mean, calculated as (SimK+TK)/2; the regression slope and its 95% confidence interval (CI) were reported. A two-step approach was employed to identify factors independently associated with the difference between SimK and TK. First, Pearson’s correlation analysis was conducted to screen candidate parameters, including age, gender, eye laterality, AL, CCT, ACD, LT, WTW, angle Alpha, and angle Kappa. This screening step was intended to reduce dimensionality and limit overfitting. Variables showing a significant correlation (*p* < 0.05) were retained for the next step. Second, backward stepwise multiple linear regression was performed to derive a parsimonious model by sequentially removing variables that did not reach *p* < 0.05, thereby facilitating interpretation. These variable-selection analyses were exploratory and hypothesis-generating. The initial model included age and gender regardless of their screening *p*-value because of their established clinical relevance, together with any other variables that were significant in the first screening step. A *p*-value < 0.05 was required for retention in the final model. Categorical variables were entered as dummy variables, with male and right eye as the reference categories: gender was coded as 0 = male and 1 = female, and eye laterality was coded as 0 = right eye and 1 = left eye. Model diagnostics were performed.

## 3. Results

### 3.1. Patients’ Baseline Characteristics

The study included two distinct groups for separate analysis: 932 eyes with prior myopic LASIK, and 372 eyes with prior RK. The baseline characteristics of both groups are summarized in Table 1.

### 3.2. Keratometry Measurements

The mean and median values of SimK and TK in eyes with prior myopic LASIK and those with prior RK are presented in Table 2. There were significant systematic differences between SimK and TK in both groups. Specifically, in eyes with prior myopic LASIK, SimK was significantly higher than TK, with a mean difference of +0.61 D (95% CI, +0.60 to +0.63 D; paired *t*-test, *p* < 0.001). In contrast, in eyes with prior RK, SimK was significantly lower than TK, with a mean difference of −0.13 D (95% CI, −0.15 to −0.11 D; paired *t*-test, *p* < 0.001).

### 3.3. Agreement Between SimK and TK

**Figure 2:**
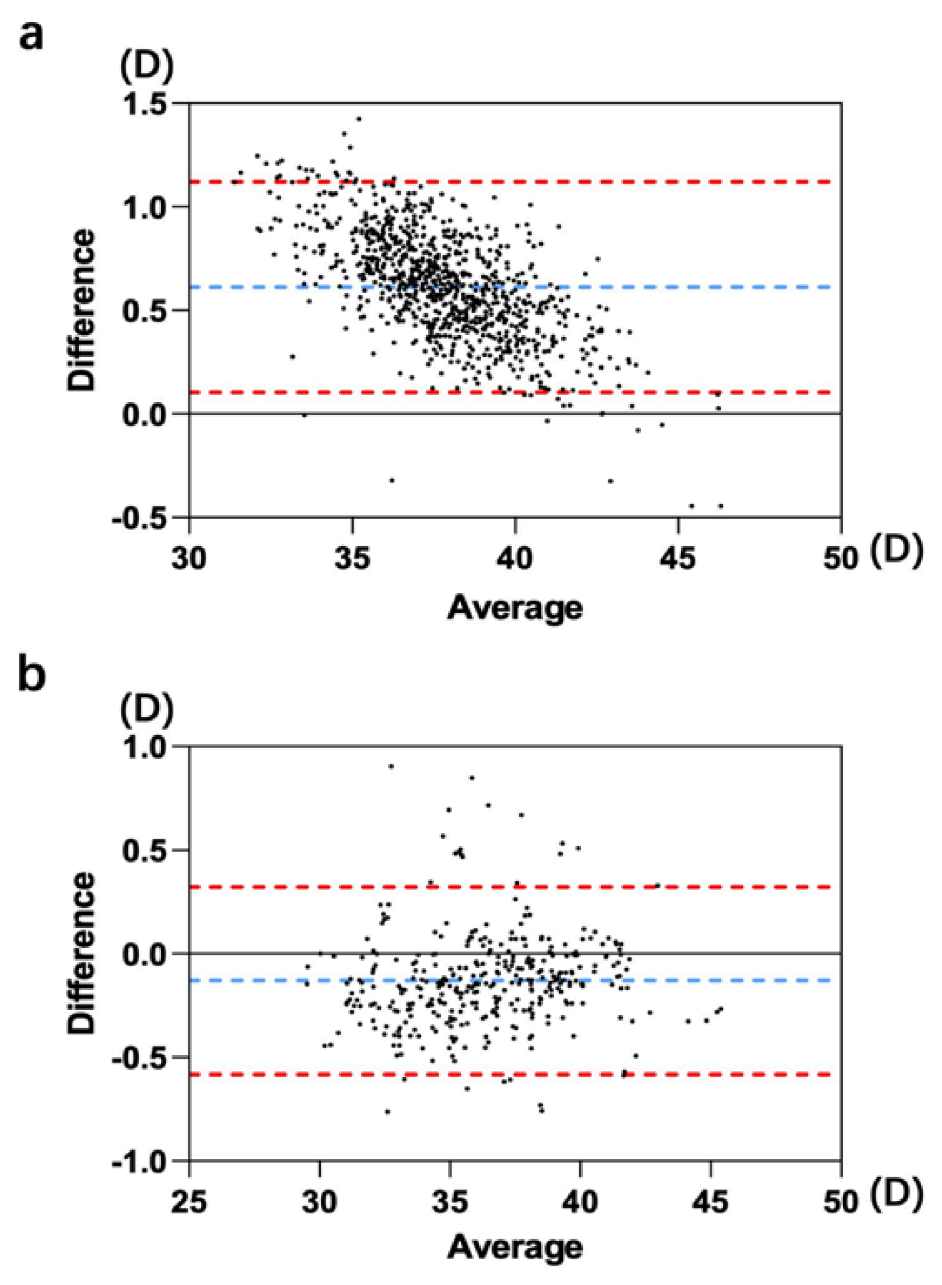
Assessment of agreement between SimK and TK. Bland–Altman plots display the differences between SimK and TK (SimK−TK) versus their average in (**a**) eyes with prior myopic LASIK and (**b**) those with prior RK. The central blue dashed line and the upper and lower red dashed lines represent the mean bias and the 95% limits of agreement (LoA), respectively.

SimK and TK were strongly positively correlated in eyes with prior myopic LASIK and those with prior RK (Pearson’s correlation analysis; r = 0.996 and 0.997, respectively; both *p* < 0.001). Bland–Altman analysis was performed to assess the agreement between SimK and TK in each group (Figure 2). In eyes with prior myopic LASIK (Figure 2a), the mean bias (SimK−TK) was positive (+0.61 D). The linear regression of the difference (SimK−TK) on the average keratometry yielded a slope of −0.074 (95% CI, −0.080 to −0.068; *p* < 0.001), indicating a statistically significant proportional bias, with the difference decreasing as average keratometry increased. The 95% limits of agreement (LoA) ranged from +0.10 to +1.12 D. Conversely, in eyes with prior RK (Figure 2b), the mean bias (SimK−TK) was negative (−0.13 D), with 95% LoA ranging from −0.58 to +0.32 D. The regression slope for proportional bias was 0.008 (95% CI, −0.002 to 0.018; *p* = 0.12), indicating no significant proportional bias in this group.

### 3.4. Factors Influencing the Difference

**Figure 3:**
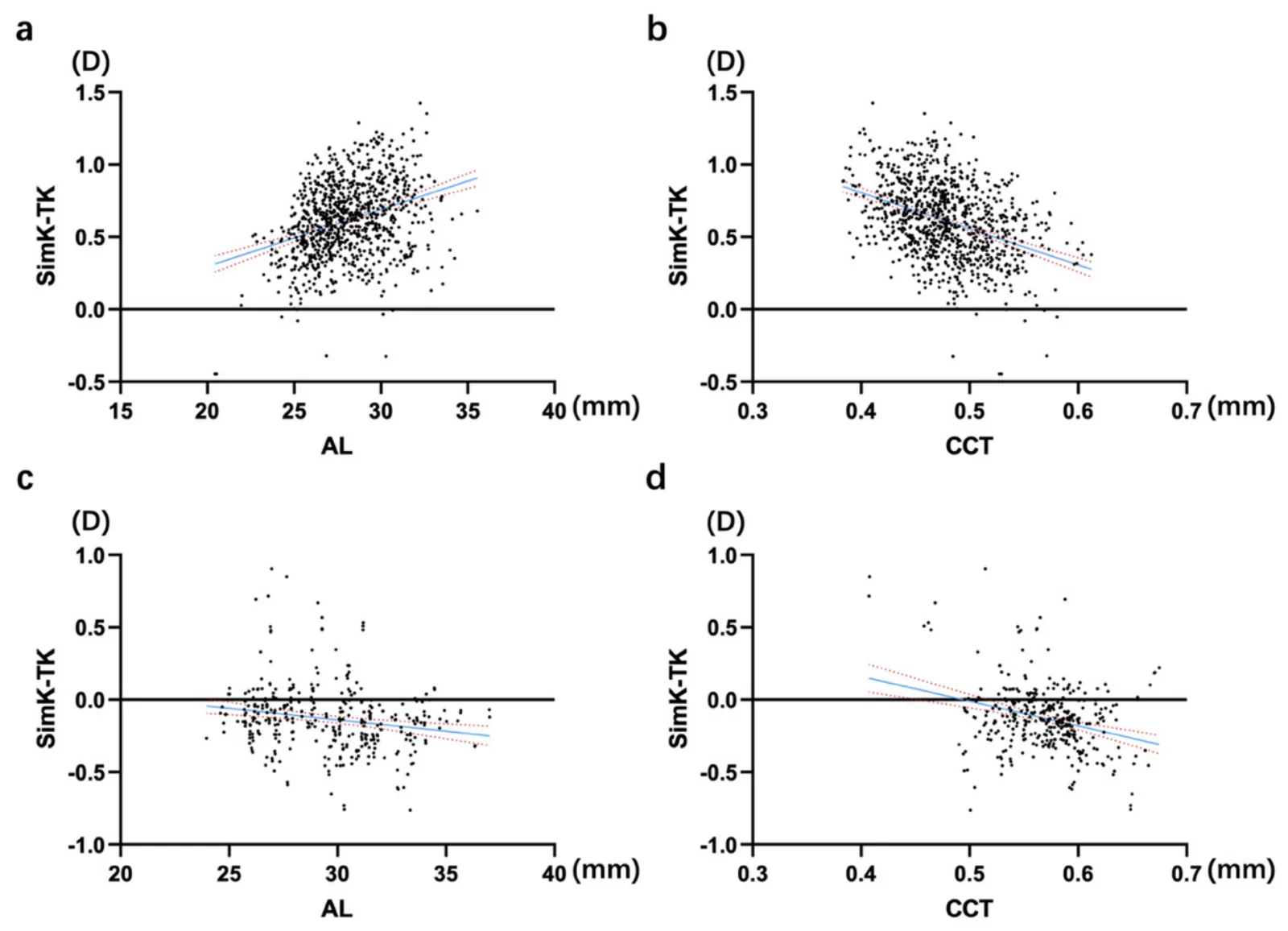
Scatterplots of the difference versus AL or CCT. The blue solid line and the red dashed lines represent the best-fit line and its 95% confidence bands, respectively. Scatterplots show the difference versus (**a**) AL in eyes with prior myopic LASIK, (**b**) CCT in eyes with prior myopic LASIK, (**c**) AL in eyes with prior RK, and (**d**) CCT in eyes with prior RK.

In eyes with prior myopic LASIK and those with prior RK, axial length (AL) and central corneal thickness (CCT) were significantly correlated with the differences between SimK and TK (Pearson’s correlation analysis; AL: r = 0.336 for LASIK, r = −0.191 for RK; CCT: r = −0.392 for LASIK, r = −0.293 for RK; all *p* < 0.001). No other significant associations were observed for the remaining parameters (age, gender, eye laterality, ACD, LT, WTW, angle Alpha, and angle Kappa). The scatterplots of the difference versus AL or CCT in both groups are shown in Figure 3.

The blue solid line and the red dashed lines represent the best-fit line and its 95% confidence bands, respectively. Scatterplots show the difference versus (a) AL in eyes with prior myopic LASIK, (b) CCT in eyes with prior myopic LASIK, (c) AL in eyes with prior RK, and (d) CCT in eyes with prior RK.

Furthermore, the backward stepwise multiple linear regression analysis demonstrated that the difference was independently associated with AL and CCT in both groups. In the LASIK group, the final model was significant (F(2, 929) = 105.525, *p* < 0.001; R^2^ = 0.185, adjusted R^2^ = 0.183); AL was positively associated with the difference (B = 0.034 D/mm, 95% CI, 0.027 to 0.040 D/mm; β = 0.346; *p* < 0.001), and CCT was negatively associated (B = −2.095 D/mm, 95% CI, −2.458 to −1.733 D/mm; β = −0.286; *p* < 0.001). In the RK group, the final model was significant (F(2, 369) = 26.088, *p* < 0.001; R^2^ = 0.124, adjusted R^2^ = 0.119); AL was negatively associated with the difference (B = −0.018 D/mm, 95% CI, −0.026 to −0.010 D/mm; β = −0.217; *p* < 0.001), and CCT was negatively associated (B = −1.600 D/mm, 95% CI, −2.152 to −1.048 D/mm; β = −0.275; *p* < 0.001). Age and gender were not retained in the final model in either group (all *p* > 0.05). Model diagnostics are summarized in Supplementary Table S1.

## 4. Discussion

The growing population of patients with prior myopic LASIK or RK complicates precise cataract surgery, primarily due to inaccuracies in keratometry measurements, a key component in IOL power calculation [21,22]. These inaccuracies stem from permanent corneal alterations induced by corneal ablation after LASIK or radial incisions after RK. However, it remains unclear how these two procedures differentially influence keratometry measurements. To address this, our study aimed to separately evaluate keratometry measurements in eyes with prior myopic LASIK and those with prior RK. We found that SimK was significantly higher than TK in eyes with prior myopic LASIK but significantly lower in eyes with prior RK. Although strongly correlated, SimK and TK demonstrated limited agreement in both groups, indicating that they should not be considered interchangeable in IOL power calculation, particularly in eyes after myopic LASIK with longer AL or thinner CCT and in those after RK with shorter AL or thinner CCT. These findings help clarify the relationship between SimK and TK in eyes with prior myopic LASIK or RK—and how this relationship differs between the two procedures—providing a basis for further evaluation of the accuracy of SimK- and TK-based IOL power calculation.

Our central finding was the diametrically opposed relationship between SimK and TK in eyes with prior myopic LASIK versus RK, which may be related to the distinct biomechanical changes induced by each procedure. The finding that SimK was higher than TK in eyes with prior myopic LASIK may be explained by the following two possible mechanisms: First, myopic LASIK ablates the central corneal stroma to flatten the anterior surface [23]. This alters the anterior-to-posterior curvature ratio assumed in the SimK calculation [24], which would cause SimK to underestimate the negative refractive power of the posterior surface and consequently overestimate the total corneal power. Second, the ablation biomechanically weakens the cornea, which may lead to anterior bowing of the posterior surface and further increase its negative refractive power—an effect not captured by SimK [25,26,27]. In contrast, TK incorporates direct measurement of the posterior surface, allowing it to account for this enhanced negative refractive power and thus yield a lower total corneal power than SimK. Conversely, the opposite relationship observed in eyes with prior RK, where SimK was lower than TK, may reflect the unique biomechanical impact of this incisional surgery. Studies have suggested that RK induces more profound flattening in the posterior than in the anterior corneal surface [28]. This is captured by TK but not accounted for by SimK, which may explain the lower SimK than TK.

The Bland–Altman analysis further revealed a proportional bias in eyes with prior myopic LASIK, suggesting that the difference between SimK and TK increased with flatter corneas in these eyes. A plausible explanation is that eyes with higher myopic correction tend to exhibit greater discrepancy; however, detailed historical LASIK information, including preoperative corneal characteristics, the magnitude of refractive correction, the ablation profile, and time since surgery, was unavailable. Beyond this LASIK-specific proportional bias, the agreement between SimK and TK was limited in both groups: the 95% LoA ranged from +0.10 to +1.12 D for LASIK and from −0.58 to +0.32 D for RK. Notably, although SimK and TK were strongly correlated, the wide LoA indicated considerable interindividual variation, highlighting that a high correlation does not ensure individual-level agreement. Therefore, SimK and TK should not be considered interchangeable in clinical practice, and the choice of keratometry values requires careful individual assessment in eyes with prior myopic LASIK or RK.

Our analysis revealed that the difference between SimK and TK was significantly associated with axial length (AL) and central corneal thickness (CCT) in both groups. In eyes with prior myopic LASIK, the difference increased with longer AL and thinner CCT, possibly reflecting that the inherent limitations of the SimK calculation might be amplified in more myopic eyes with greater corneal ablation. Conversely, in eyes with prior RK, the difference increased with shorter AL and thinner CCT. We speculated that this could be attributed to the complexity and unpredictability of RK surgery. Eyes with shorter AL or possibly more aggressive incisions (indirectly reflected by thinner CCT) might have been associated with a greater hyperopic shift after RK [29,30], potentially corresponding to the larger keratometry difference observed in these eyes. Importantly, these interpretations remain speculative, as key historical surgical parameters—including the magnitude of the original refractive correction, preoperative keratometry, time since surgery, LASIK treatment characteristics, and details of RK incisions—were not available for either group. Further studies with complete surgical data are needed to confirm these mechanisms and clarify their clinical relevance.

Beyond quantifying the discrepancy, our findings have potential clinical implications. First, the surgery-specific direction of the systematic bias (SimK > TK after LASIK versus SimK < TK after RK) may provide a useful clue in eyes with an incomplete medical record or an unclear history of corneal refractive surgery. However, this potential application requires further validation in eyes with an uncertain or combined surgical history. In particular, whether the direction and magnitude of the difference between SimK and TK can help distinguish between prior myopic LASIK and RK, or inform the selection of surgery-specific IOL calculation formulas, warrants further investigation [31,32].

This study has several limitations that should be acknowledged. First, this was a single-center retrospective study of a selected high-myopia population with relatively long axial lengths; the findings may therefore not generalize to the broader population of cataract patients with prior LASIK or RK. Second, historical refractive-surgery information (original refractive correction, preoperative keratometry, time since surgery, LASIK treatment characteristics, and RK incision details) was incomplete, precluding adjustment for these factors and limiting the mechanistic interpretations to hypotheses. Third, postoperative refractive outcomes were unavailable, so the clinical impact on IOL prediction could not be assessed. Fourth, no consensus clinical agreement threshold currently exists for SimK and TK; Bland–Altman results were therefore reported descriptively, and interchangeability was assessed based on the observed LoA and proportional bias. Finally, measurement repeatability was not formally assessed due to the variability inherent in routine clinical practice.

In conclusion, our study identified systematic differences between SimK and TK by IOLMaster 700 in eyes with prior myopic LASIK and RK. Specifically, in eyes with prior myopic LASIK, SimK was significantly higher than TK. However, in eyes with prior RK, SimK was significantly lower than TK. Despite the strong correlation, the limited agreement between SimK and TK suggests that they should not be considered interchangeable in these eyes, particularly in those after LASIK with longer AL or thinner CCT and those after RK with shorter AL or thinner CCT. Therefore, we suggest that clinicians account for these systematic biases when selecting between SimK and TK in eyes with prior myopic LASIK or RK, pending further validation of the clinical impact on IOL calculation and surgical outcomes.

## Figures and Tables

**Table 1:** Baseline characteristics.

Characteristics	LASIK	RK
Age (years)	48.1 ± 10.3	56.8 ± 8.8
Gender, *n* (%)		
Male	367 (39.4)	159 (42.7)
Female	565 (60.6)	213 (57.3)
Eye laterality, *n* (%)		
Right eye	448 (48.1)	183 (49.2)
Left eye	484 (51.9)	189 (50.8)
Axial length (mm)	28.04 ± 2.22	29.47 ± 2.79
Central corneal thickness (mm)	0.48 ± 0.04	0.57 ± 0.04
Anterior chamber depth (mm)	3.40 ± 0.34	3.50 ± 0.51
Lens thickness (mm)	4.18 ± 0.40	4.31 ± 0.47
White-to-white (mm)	11.86 ± 0.41	11.89 ± 0.44
Angle Alpha (mm)	0.29 ± 0.15	0.37 ± 0.20
Angle Kappa (mm)	0.27 ± 0.16	0.36 ± 0.21

**Table 2:** Keratometry measurements in eyes with prior myopic LASIK and those with prior RK.

	SimK (D)		TK (D)	
	Mean ± SD	Median (IQR)	Mean ± SD	Median (IQR)
LASIK	38.20 ± 2.19	38.08 (36.76, 39.61)	37.59 ± 2.36	37.51 (36.09, 39.07)
RK	36.40 ± 2.98	36.48 (34.41, 38.42)	36.53 ± 2.96	36.56 (34.50, 38.59)

SD, standard deviation; IQR, interquartile range.

## Data Availability

The datasets used and/or analyzed during the current study are available from the corresponding author on reasonable request.

## Supplementary Materials

The following supporting information can be downloaded at: https://www.mdpi.com/article/10.3390/jcm15218749/s1, Supplementary Table S1. Regression model diagnostics.
