# Supplementary material for: Simulated Keratometry Versus Total Keratometry: Separate Evaluations in 932 Eyes with Prior Myopic LASIK and 372 Eyes with Prior Radial Keratotomy
Source: J Clin Med. 2026 Nov 9;15(21):8749. doi: 10.3390/jcm15218749 (PMC13763622; doi:10.3390/jcm15218749)
Supplement: Supplementary file 1 [file jcm-15-08749-s001.zip › jcm-4613374-supplementary.pdf]

Supplementary Table S1 Regression model diagnostics

Diagnostic	LASIK	RK
Maximum VIF	1.046	1.035
Residual skewness	-0.16	0.66
Residual excess kurtosis	0.09	1.74
Std. residual range	-2.39 to 2.92	-2.90 to 2.72
Maximum Cook’s D	0.046	0.172

VIF, variance inflation factor; Std. residual, standardized residual; Cook’s D, Cook’s distance. Maximum VIF was 1.046 in the LASIK group and 1.035 in the RK group, indicating negligible multicollinearity. Residual skewness and excess kurtosis were used to assess departure from normality; standardized residuals ranged from -2.39 to 2.92 in the LASIK group and from -2.90 to 2.72 in the RK group; no standardized residual exceeded an absolute value of 3. Maximum Cook’s D was 0.046 in the LASIK group and 0.172 in the RK group; no observation exceeded the conventional Cook’s D threshold of 1.
